# Cannabidiol for the Treatment of Neonatal Hypoxic-Ischemic Brain Injury

**DOI:** 10.3389/fphar.2020.584533

**Published:** 2021-01-11

**Authors:** José Martínez-Orgado, María Villa, Aarón del Pozo

**Affiliations:** ^1^ Division of Neonatology, Hospital Clínico San Carlos – IdISSC, Madrid, Spain; ^2^ Biomedical Research Foundation Hospital Clinico San Carlos, Madrid, Spain

**Keywords:** cannabidiol, hypoxia-ischemia, neuroprotection, brain, newborn

## Abstract

Each year, more than two million babies die or evolve to permanent invalidating sequelae worldwide because of Hypoxic-Ischemic Brain Injury (HIBI). There is no current treatment for that condition except for therapeutic hypothermia, which benefits only a select group of newborns. Preclinical studies offer solid evidence of the neuroprotective effects of Cannabidiol (CBD) when administered after diffuse or focal HI insults to newborn pigs and rodents. Such effects are observable in the short and long term as demonstrated by functional, neuroimaging, histologic and biochemical studies, and are related to the modulation of excitotoxicity, inflammation and oxidative stress—the major components of HIBI pathophysiology. CBD protects neuronal and glial cells, with a remarkable effect on preserving normal myelinogenesis. From a translational point of view CBD is a valuable tool for HIBI management since it is safe and effective. It is administered by the parenteral route *a posteriori* with a broad therapeutic time window. Those findings consolidate CBD as a promising treatment for neonatal HIBI, which is to be demonstrated in clinical trials currently in progress.

## Hypoxic-Ischemic Brain Injury in Newborns

Diffuse or focal acute hypoxic-ischemic brain injury (HIBI) is a prevalent condition affecting 1 to 9 out of 1000 live newborns (Martínez-Orgado, 2014). As far as focal HIBI is concerned, incidence in the neonatal period is in fact as high as in adulthood (Kratzer et al., 2014). In global terms nearly 2 million babies die or remain with long-lasting detrimental consequences, including motor and cognitive deficits each year (Martínez-Orgado, 2014; Parikh and Juul, 2018). Thus, neonatal HIBI is the main known cause of Cerebral Palsy, a devastating non-progressive degenerative disorder that compromises the lives of children and families and represents a tremendous socio-economic burden on society (Nelson, 2008). Those figures have not changed substantially in the last few years because clinical management of neonatal HIBI is challenging. On the one hand, the complex pathophysiology of HIBI implies that only therapies encompassing multiple mechanisms can be really effective (Juul and Ferriero, 2014; Linsell et al., 2016; Parikh and Juul, 2018). On the other hand, early treatment is hardly achievable. In the case of diffuse HIBI although the neurologic condition derived from this situation, known as neonatal hypoxic-ischemic encephalopathy (NHIE) has a well-characterized clinical picture, determining the exact moment when the HI insult took place before delivery is very difficult (Gonzalez and Ferriero, 2008). In the case of focal HIBI, Perinatal Arterial Ischemic Stroke (PAIS), the clinical picture is so subtle that less than 25% of cases are diagnosed immediately (Armstrong-Wells and Ferriero, 2014). Therefore, neuroprotective therapies aiming to reduce HIBI should be effective when administered *a posteriori* to the HI event and show a broad therapeutic time window (TTW) (Gonzalez and Ferriero, 2008). Finally, treatments to be administered to newborns must not only be free from serious side effects in the short-term but free from detrimental effects on development as well (Juul and Ferriero, 2014; Parikh and Juul, 2018).

**Figure 1:**
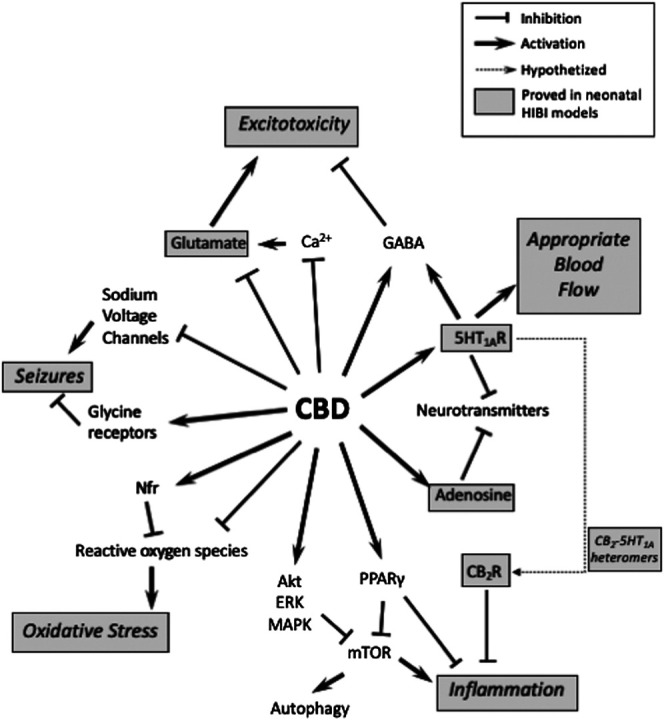
Schematic representation of different molecular targets of cannabidiol (CBD) and their involvement on the mechanisms of hypoxic-ischemic brain injury (HIBI). Dot-lined boxes indicate which ones have been studied in preclinical models of neonatal HIBI.

### Pathophysiology of Hypoxic-Ischemic Brain Injury in Newborns

Immature brain is particularly susceptible to HIBI mainly because of high metabolic rate, immature cerebral blood flow autoregulation mechanisms, paucity of anti-oxidant defenses, high density and sensitivity of receptors to excitatory amino acids such as glutamate and high sensitivity to inflammation (Martínez-Orgado et al., 2007; Johnston et al., 2011; Rainaldi and Perlman, 2016). Energy failure during HI causes dysfunction of ionic pumps leading to changes in membrane potential, deregulation of ion homeostasis and glutamate excitotoxicity, which triggers increased intracellular calcium levels that activates different enzymes involved in neuronal cell death including caspases, lipases, endonucleases and nitric oxide production (Martínez-Orgado et al., 2007; Johnston et al., 2011; Rainaldi and Perlman, 2016). During post-ischemic reperfusion, inflammation and exacerbated oxidative stress increase and spread neuron and glial cell damage (Martínez-Orgado et al., 2007; Johnston et al., 2011; Rainaldi and Perlman, 2016). In the immature brain, the initial HI insult is followed by a latent period lasting 6–24 h, during which excitotoxicity, inflammation and oxidative stress act to induce the development of cell events which lead to a secondary deterioration because of delayed energy failure (Gonzalez and Ferriero, 2008; Johnston et al., 2011; Parikh and Juul, 2018). This latent phase offers a window of opportunity for treatment with neuroprotectants. Although apoptotic and necrotic cell death are a continuum in immature brain after HI, apoptosis plays a particularly relevant role in neonatal HIBI pathophysiology (Rocha-Ferreira and Hristova, 2016). In addition, immature brain is characterized by a greater impact of autophagy-related cell death after HI insults than adult brain (Descloux et al., 2015).

Glial cells play a key role in HIBI pathophysiology. Immature oligodendroglial cells are extremely vulnerable to inflammatory, excitotoxicity and oxidative damage (Back et al., 2002), which results in extensive myelination disturbance leading to long-lasting motor, sensorial and cognitive disabilities (Nelson, 2008; Volpe, 2010). Astrocytes are essential to support the neurons that survive acute HI damage, attenuate oxidative stress and glutamate excitotoxicity, release neurotrophic factors and preserve the integrity of the blood-brain barrier thereby limiting brain invasion by inflammatory cells during reperfusion (Takuma et al., 2004; Barreto et al., 2011). Increased population of astrocytes, known as astrogliosis, is a well-known long-term marker of HIBI due to the formation of a glial scar in areas of infarct (Colangelo et al., 2014). However, shortly after the HI insult activation and reduction of astrocyte population corresponds to the severity of HIBI since such a response is related to excitotoxic damage of astrocytes which implies blunting of its homeostatic role (Takuma et al., 2004; Barreto et al., 2011; Mallard et al., 2014). HI insults lead to a greater increase of microglia and more robust expression of pro-inflammatory cytokines in activated microglia -the M1 phenotype-in immature than in mature brain (Ferrazzano et al., 2013), although the protective effects of the anti-inflammatory M2 microglial phenotype is also particularly relevant in immature brain (Mallard et al., 2014). The increasing importance paid to the interrelated, complex and time-dependent roles that astrocytes and microglial cells play in neonatal HIBI makes glial cell protection from HI injury a critical component of neuroprotective strategies (Mallard et al., 2014).

### Treatment

Therapeutic hypothermia (TH) became the standard of care to treat newborns with NHIE after revealing an improved outcome, which reduces death and/or severe disability in the long term (Natarajan et al., 2016; Parikh and Juul, 2018). The total tissue water (TTW) for TH is 6 h, although TH initiated between 6 h and 24 h after birth may have some beneficial effects (Laptook et al., 2017). However, TH efficacy has been revealed mostly in mild NHIE, in babies over 35 weeks of gestational age at birth and when provided in selected centers in developed countries (Natarajan et al., 2016; Parikh and Juul, 2018). In addition to those limitations, an unacceptable 40%–50% of NHIE patients eligible to receive TH still show no benefits from the treatment (Parikh and Juul, 2018). Thus, synergistic or complementary therapies to hypothermia are warranted. Different strategies have been tested in the experimental setting such as melatonin, erythropoietin, xenon or stem cells (Parikh and Juul, 2018) but they have not demonstrated clinical benefits so far.

There is no specific treatment for PAIS (Armstrong-Wells and Ferriero, 2014). In preclinical studies melatonin or erythropoietin are effective on reducing brain damage, whereas hypothermia offers just mild and contradictory results (Harbert et al., 2011; Villapol et al., 2011; Gonzalez et al., 2013).

## Cannabidiol for Neuroprotection in Neonatal Hypoxic-Ischemic Brain Injury

CBD has a complex poly-pharmacologic profile and regulates the activity of different signaling proteins and receptors acting on various molecular targets (Izzo et al., 2009; Campos et al., 2017). In different experimental paradigms CBD shows robust anti-oxidant and anti-inflammatory activity, inhibits calcium flux across membranes, inhibits endocannabinoid uptake and enzymatic hydrolysis, reduces glutamate release, stabilizes the mitochondrial membrane, regulates different receptor types including serotonin 5HT_1A_ and PPARγ receptors, augments the extracellular concentration of adenosine and prevents NF-κB activation (Pertwee, 2004; Mechoulam et al., 2007). All those effects (Figure 1) account for a potential neuroprotective efficacy and prompted the study of CBD as a neuroprotectant for neonatal HIBI.

### Europrotective Efficacy of Cannabidiol in Experimental Models of Hypoxic-Ischemic Brain Injury

Incubation of newborn mice forebrain slices *in vitro* exposed to oxygen-glucose deprivation (OGD) with CBD 100 µM reduces LDH release and caspase 9 expression, which indicates reduced necrotic and apoptotic cell death (Castillo et al., 2010).

CBD has been tested *in vivo* in different experimental paradigms of diffuse neonatal HIBI. In the model most widely used for that purpose, known as the Rice-Vannucci model and consisting of unilateral carotid artery clamp plus exposure to 8%–10% O_2_ in seven-to-ten-day-old (P7-P10) rodents, post-insult administration of CBD 1 mg/kg i.p. reduces the volume of damage in rats (Pazos et al., 2012) and mice (Mohammed et al., 2017). Those neuroprotective effects are sustained in the long term and associated with remarkable neurofunctional benefits; with HI rats treated with CBD after the HI insult showing normal motor and cognitive performance when they become adults (Pazos et al., 2012). In newborn piglets studied for 6 h–72 h after being exposed to moderate HI insult by temporary bilateral carotid artery occlusion and exposure to 10% O_2_, post-insult administration of CBD 0.1–1 mg/kg i.v. restores brain electrical activity and reduces seizure burden as assessed by continuous EEG monitoring. This also preserves regional cerebral blood flow as assessed by near-infrared spectroscopy (NIRS), prevents the increase of lactate/N-acylaspartate (Lac/NAA) ratio thought to be the most predictive early biomarker of a poor outcome to infant HI and a surrogate endpoint used to assess neuroprotective strategies (Pazos et al., 2013); as assessed by proton magnetic resonance spectroscopy (H + -MRS) and restoring motor and behavioral performance (Alvarez et al., 2008; Lafuente et al., 2011; Pazos et al., 2013; Arruza et al., 2017). However, when piglets were exposed to very severe HI insults CBD administration does not lend neuroprotection (Garberg et al., 2016; Barata et al., 2019). In this case TH administered to the piglets was also ineffective (Garberg et al., 2016; Barata et al., 2019).

Noteworthy, CBD neuroprotection remains unaffected in spite of delaying administration of CBD up to 18 h after the HI insult in newborn mice, a TTW broader than that of TH and other neuroprotective substances (Mohammed et al., 2017).

Regarding focal neonatal HIBI, CBD efficacy was studied using a model of Middle Cerebral Artery Occlusion (MCAO) adapted to newborn rats. In this model of PAIS post-insult administration of CBD 3 mg/kg reduces the volume of infarct and restores motor and cognitive performance in the long term (Ceprián et al., 2017).

### Effects of Cannabidiol on Neuron and Glia Damage

Since glioprotection is now considered as important as neuroprotection to reduce HIBI (Mallard et al., 2014; Parikh and Juul, 2018) it is important to remark that CBD has demonstrated protection of both neuronal and glial cells in models of HIBI.

Post-HI administration of CBD to piglets reduces neuronal death, as reflected by the prevention of HI-induced increase on cerebrospinal fluid concentration of neurospecific enolase (Lafuente et al., 2011) in association with the prevention of necrotic and apoptotic neuronal death, as observed in parietotemporal cortex 6 h and 72 h after the end of the HI insult by Nissl and TUNEL staining and caspase 3 concentration measurement by Western blot analysis (Alvarez et al., 2008; Lafuente et al., 2011; Lafuente et al., 2016; Pazos et al., 2013; Arruza et al., 2017; Barata et al., 2019). In newborn rodents CBD reduction of diffuse HI-induced neuronal death in the cortex is significant but less dramatic, although the effect is still observable when animals become adults (Pazos et al., 2012) and includes the modulation of apoptotic death (Mohammed et al., 2017). In the PAIS model in newborn rats CBD fully prevents stroke-induced reduction of the neuronal population and increased density of TUNEL+ neurons in the cortex (Ceprián et al., 2017).

Treatment of HI piglets with CBD results in the prevention of HI-induced astrocyte activation and population reduction as assessed by immunohistochemistry GFAP labelling in the cortex as well as cerebrospinal fluid S100ß protein concentration measurement (Lafuente et al., 2011; Pazos et al., 2013). In addition, CBD administration to HI newborn rats reduces the severity of long-term astrogliosis (Mohammed et al., 2017). In focal HIBI, in addition to the reduction of long-term astrogliosis early post-stroke protection of astrocyte integrity by CBD is demonstrated by the prevention of ischemic-induced reduction of myoinositol/creatine ratio, a marker of cytolytic astrocyte dysfunction, as assessed by H^+^-MRS (Ceprián et al., 2017).

In HI piglets, 6 h after diffuse HI insult CBD does not reduce the number of microglial cells in the cortex but modifies the proportion of M1 and M2 phenotypes, which reduces the presence of the pro-inflammatory M1 phenotype (Barata et al., 2019). Since microglial activation leads to increased microglial proliferation, the fact that in newborn rats exposed to diffuse HI insult CBD prevents HI-induced proliferation of microglial cells in the cortex as assessed seven days post-insult is not surprising (Mohammed et al., 2017). After focal HIBI in newborn rats, CBD reduction of microglial activation and subsequent proliferation is even more remarkable 30 days post-stroke, which indicates that modulation of microglial activation by CBD is sustained in the long term (Ceprián et al., 2017).

CBD administration to newborn rats after diffuse HIBI preserves normal myelination. Thus, in HI rats studied 30 days after the insult CBD prevents HI-induced reduction in the number of mature oligodendrocytes and myelin basic protein signal as assessed by immunohistochemistry in the cortex and White Matter, as well as HI-induced reduction of axonal density and myelin sheath thickness as assessed by Electronic Microscopy in the same areas (Ceprián et al., 2019). This reasonably accounts for the fact that CBD treatment is more effective in restoring neurobehavioral function than in reducing the volume of brain damage (Pazos et al., 2012). Post-insult administration of a treatment implies that a substantial amount of brain tissue is irreversibly damaged by HI at the time the treatment is administered. However, preserving normal myelination in the perilesional surviving tissue is the basis for the brain to develop compensatory mechanisms to eventually attain normal function. There are no reports of the effects of CBD treatment on myelination after stroke in newborn animals.

### Mechanisms of Action of Cannabidiol

CBD acts on the main factors leading to cell death in HIBI: excitotoxicity, oxidative stress and inflammation.

*In vitro*, incubation of newborn mice forebrain slices exposed to OGD with CBD dramatically reduces the increase in glutamate release observed in the first 30 min after the insult (Castillo et al., 2010). *In vivo*, CBD treatment fully prevents HI-induced increase of glutamate/N-acylaspartate (Glu/NAA) ratio in the brain -which is proportional to the severity of encephalopathy in human newborns (Pazos et al., 2013)- as assessed in piglets 6 h after a moderate HI insult by H^+^-MRS studies (Pazos et al., 2013; Lafuente et al., 2016). This effect is still observable in newborn rats seven days after the HI insult (Pazos et al., 2012) but not in piglets three days after a very severe HI insult (Barata et al., 2019).

In *in vivo* studies in newborn piglets CBD prevents HI-induced increase in brain concentration of MDA (Lafuente et al., 2011) as well as HI-induced consumption of reduced glutathione 6 h after the HI insult (Pazos et al., 2013). This effect is still observable seven days after the HI insult in newborn rat brain (Pazos et al., 2012). In newborn piglets CBD prevents HI-induced increase of protein carbonylation, a specific marker of increased oxidative stress involved in HIBI pathophysiology (Pazos et al., 2013; Lafuente et al., 2016; Arruza et al., 2017). However, to determine the real efficacy of CBD against HI-induced oxidative stress in HIBI, further research is warranted about the effects of CBD on brain neuroprostane or neurofurane concentration as reliable biomarkers of neuronal lipid peroxidation after HI (Garberg et al., 2016).

Given the modulatory effect of CBD on microglial activation CBD was also expected to moderate the release of pro-inflammatory cytokines. *In vitro,* incubation of newborn mice forebrain slices exposed to OGD with CBD prevents post-insult increase of IL-1a and TNFa concentration as well as COX-2 expression (Castillo et al., 2010). *In vivo*, CBD administration after moderate HI insult in newborn piglets prevents the increased brain concentration of those cytokines as studied by Western blot or microarrays six (Pazos et al., 2013; Lafuente et al., 2016; Arruza et al., 2017) or 72 h (Lafuente et al., 2011) after the insult. CBD prevention of HI-induced increased brain TNFa concentration can still be observed seven days after the HI insult in newborn rats (Pazos et al., 2012). By contrast, CBD administration to newborn piglets after a very severe HI insult does not reduce HI-induced increase in brain TNFa concentration (Barata et al., 2019). It is known that PPARγ activation plays a key role in the anti-inflammatory effects of CBD (Esposito et al., 2011) but this has not been studied in models of neonatal HIBI. This point is of interest since PPARγ activation is involved in CBD inhibition of mTOR, the main activator of autophagy which plays a relevant role in neonatal HIBI pathophysiology (Galluzzi et al., 2016).

Modulation of those mechanisms is likely key to CBD neuroprotection, since prevention of cell loss by CBD in immature rat brain after HI insults is not associated with increased expression of neuroproliferative factors such as BDNF or GDNF (Ceprián et al., 2019).

CBD is a 5HT_1A_ receptor agonist and inhibits 5HT re-uptake (Russo et al., 2005). In newborn piglets exposed to moderate HI insult, administration of a 5HT_1A_ receptor antagonist together with CBD eliminates all the beneficial effects of CBD (Pazos et al., 2013), which supports the key role of 5HT_1A_ activation in the neuroprotective effects of CBD in immature brain. CBD is traditionally known to not act through CB_1_ or CB_2_ receptor activation (Pertwee, 2004; Mechoulam et al., 2007). Accordingly, blockade of CB_1_ receptors does not modify CBD effects in mice forebrain slices exposed to OGD (Castillo et al., 2010). The role of CB_2_ receptors on CBD neuroprotection in immature brain, however, is more controversial. Coincubation with CB_2_ antagonists reversed all the neuroprotective effects of CBD in mice forebrain slices exposed to OGD (Castillo et al., 2010). Similarly, administration of CB_2_ antagonists together with CBD to newborn piglets after HI insult attenuated all the neuroprotective effects of CBD (Pazos et al., 2013). Since it has been repeatedly demonstrated that CBD does not act as an activator of CB_2_ receptors, those effects could be accounted for by an indirect cross-activation of CB_2_ receptors in CB_2_-5HT_1A_ heteromers. CB_2_-5HT_1A_ heteromers are present and functioning in rat brain. Their density is higher in immature than in mature brain and increased after HI insults particularly in immature brains (Franco et al., 2019). *In vitro*, antagonism of adenosine A_2A_ receptors blocked the neuroprotective effects of CBD in newborn mice forebrain slices exposed to OGD, suggesting that A_2A_ receptors are involved in CBD neuroprotection in immature brain, with a particular relevance on the anti-apoptotic effects of CBD (Castillo et al., 2010). The involvement of other receptors known to be a target for CBD, such as PPARγ, GPR55 or TRPV1 (Pertwee, 2004), has not yet been explored in models of neonatal HIBI.

CBD inhibits endocannabinoid uptake and enzymatic hydrolysis *in vitro* (Pertwee, 2004; Mechoulam et al., 2007). This appears to be of marginal importance at least in the early moments after HI insults in newborn brain since in fact CBD administration prevents HI-induced increase in endocannabinoid concentration observed in piglet brain 6 h after insult (Pazos et al., 2013).

### Cannabidiol and Therapeutic Hypothermia

Since TH is the standard of care for asphyxiated newborns, studying how CBD can substitute or collaborate with TH is mandatory.

In newborn piglets exposed to diffuse HI insult, CBD and TH show a similar neuroprotective profile. When the HI insult is moderate, either CBD or TH similarly prevent HI-induced increase in neuronal loss, Lac/NAA ratio, caspase 3 expression, excitoxicity, inflammation and oxidative stress as assessed 6 h after the insult (Lafuente et al., 2016). When the HI insult is severe, neither 48 h-long TH nor CBD 1 mg/kg/d for three days are able to prevent HI-induced increased apoptosis, Lac/NAA ratio, excitotoxicity and cytokine concentration (Garberg et al., 2016; Barata et al., 2019). Noteworthy, even under those circumstances CBD exerts a modulatory effect on microglial activation, an effect not observable in piglets treated with TH (Barata et al., 2019).

When administered in combination after a moderate HI insult in newborn piglets, CBD and TH reveal additive effects, with the combination of both therapies leading to better neuroprotective effects than CBD or TH alone (Lafuente et al., 2016). However, combining CBD with TH in piglets exposed to severe HI insult led to controversial results depending on the experimental model. In piglets exposed to severe anoxia and systemic hypotension the combination of CBD and TH does not result in additive affects (Garberg et al., 2016). By contrast, in piglets exposed to severe hypoxia and brain ischemia combining CBD and TH has synergistic effects, because if CBD or TH alone are not protective, the combination of both therapies effectively reduces apoptotic death, Lac/NAA increase, inflammation and excitotoxicity as assessed in brain cortex three days after the HI insult (Barata et al., 2019).

### Pharmacologic Aspects of Cannabidiol in Neonatal Hypoxic-Ischemic Brain Injury

Although CBD is a lipid substance, a formulation of CBD in saline, ethanol and a solvent as solutol or cromophor is suitable for parenteral administration (Alvarez et al., 2008; Lafuente et al., 2011; Pazos et al., 2012; Pazos et al., 2013; Lafuente et al., 2016; Ceprián et al., 2017; Barata et al., 2019; Ceprián et al., 2019). PK studies in newborn piglets receiving 1 mg/kg i.v and using that formulation indicate that plasma CBD concentration peaks by 15 min after the end of the infusion and attains 200–300 ng/ml. It is nearly undetectable 12 h post infusion, with t_1/2_ approximately 2 h (Barata et al., 2019). CBD administered in this way to HI piglets attains a brain concentration of about 60 ng/g 6 h post-infusion (Pazos et al., 2013; Barata et al., 2019), which is equivalent to 200 nM. In newborn rats receiving CBD at the same dose using the same formulation i.p. brain concentration peaked 3 h post administration reaching about 30 ng/g. It was still detectable 36 h post administration (Pazos et al., 2012). Studies in piglets of CBD and TH revealed that hypothermia did not modify plasma or brain concentration of CBD or its metabolites, with the exception of a mild increase in the very low plasma levels of 6-OH-CBD in piglets receiving CBD and TH (Barata et al., 2019).

CBD administration to HI newborn piglets is not only free from significant side effects but is associated with hemodynamic and respiratory benefits. In HI piglets, CBD prevents HI-induced myocardial troponin increase and hypotension (Alvarez et al., 2008; Pazos et al., 2013), the latter effect even more remarkable in HI piglets receiving TH (Barata et al., 2019). CBD treatment prevents the ventilatory deterioration observed in newborn piglets in the hours following the HI insult (Alvarez et al., 2008; Pazos et al., 2013; Arruza et al., 2017), an effect attributed to the prevention of distant inflammatory lung damage induced by HIBI (Arruza et al., 2017). In newborn rats CBD administration has no effects on growth, brain volume or neurobehavioral performance as assessed when rats become adults (Pazos et al., 2012).

## Conclusion

Preclinical studies offer solid evidence of the neuroprotective efficacy of CBD to limit HIBI in newborns. CBD prevents the functional deficits appearing after neonatal HIBI, which reduces the extent of brain damage and protects myelinogenesis. From a translational point of view CBD is a valuable tool for HIBI management since it is safe and effective and administered by the parenteral route *a posteriori*, with a broad therapeutic time window. CBD shows a similar neuroprotective profile as TH, which augments its efficacy when administered in combination. The fact that combining CBD and TH affords neuroprotection in severe cases of HIBI after they were ineffective when administered separately offers a promising opportunity to give some hope to the high number of asphyxiated babies showing no benefits with TH. Accordingly, a clinical trial testing CBD in asphyxiated infants is now underway (GWEP1560, EudraCT 2016-000936-17).

Although it is clear that CBD protects neuronal and glial cells by modulating key factors leading to HIBI such as excitotoxicity, oxidative stress and inflammation, further information is needed about the ultimate mechanisms of CBD neuroprotection in neonatal HIBI and how they can be determined by developmental status. At least in newborn piglets CBD could act as a substitute for TH. When combined with TH, CBD could lend neuroprotection in some cases in which TH alone is ineffective.

## Author Contributions

MV and AP participated in many of the experiments described in the text and participated in the writing and correction of the manuscript. JM-O was the Principal Investigator of all the experiments described in the text and wrote the manuscript.

## Funding

The works cited in this article were supported by grants from Instituto de Salud Carlos III by means of projects PI061085, PS0901900, PI12/00192, PI13/01722, PI16/00689 and PI19/00927 (Co-funded by the European Regional Development Fund/European Social Fund “A way to make Europe”/“Investing in your future”) from the Biomedicine Program, Community of Madrid (S2010/BMD- 2308) and from GW Research Ltd. (GWCRI09119).

## Conflict of Interest

JM-O had a research agreement with GW Research Ltd. from 2010 to 2019.

The remaining authors declare that the research was conducted in the absence of any commercial or financial relationships that could be construed as a potential conflict of interest.

The reviewer HL declared a past co-authorship with one of the authors JM to the handling Editor
